# An exploration of the factors influencing the local implementation of the Care Programme Approach in the provision of mental health services for clients with learning disabilities

**Published:** 2013-12-18

**Authors:** Michael Kelly

**Affiliations:** Department of Postgraduate Research, James Clerk Maxwell Building, King's College London, 57 Waterloo Road, London SE1 8WA, UK E-mail: michael.kelly@kcl.ac.uk

## Outline

Introduced by the UK Department of Health in 1990, the Care Programme Approach (CPA) was intended to be a framework through which health and social services could provide integrated care for people with mental health problems. Its purpose was to ensure that services had systematic arrangements to provide psychiatric, health and social care with arrangements in place for the ongoing provision and review of that care. Care coordinators, spanning service boundaries, were expected to oversee that process. In order to ensure that services could work together, the Health Act (1999) enabled local services to pool budgets, lead commissioning and integrate provision.

Despite repeated government efforts to support the policy, its implementation faced many problems. Alongside issues with resistance from professionals at a local level, there were also issues at the strategic level associated with the actual integration process.

Second to this, the policy did not acknowledge people who had mental health problems *and* a concurrent learning disability (dual diagnosis) until 10 years after the policy was introduced and thus these clients did not receive care through CPA as expected. With so little known about the strategic level factors affecting implementation or the actual implementation process for this particular client group, this thesis aimed to explore the factors shaping the local implementation of CPA for these clients from a strategic-level perspective.

## Introduction

A review of the literature on the implementation of CPA in the 1990s identified two key areas in which services struggled: the process of embracing change in an organisational context and the process of working in partnership across services. By reviewing the literature from these areas, a framework was created through which data could be analysed. This is known as *framework analysis* and is used to analyse data in the applied policy context.

In order for a detailed examination of the implementation process to be undertaken, a case study design was used in which there were five individual units of analysis. Data were collected through semi-structured interviews, documentary analysis and the administration of a qualitative questionnaire. Respondents were in the main health and social service employees from different hierarchical levels within their respective organisations. The response rate was 47%.

## Results and findings

The study identified six key strategic level factors which impacted the implementation process. These included a lack of finances and resources, competing priorities, incompatible information technology and information-sharing processes, organisational complexity, lack of governance and accountability arrangements and high staff turnover. Although these issues were not specific to CPA, they provided a background environment in which the policy was to be introduced and in which the implementation process was likely to struggle.

At a local service level, further issues were identified. Although these could be identified as being specific to CPA itself, they could in fact apply to other such policies where service integration is expected. These issues included problems with creating a shared vision, understanding and commitment, a lack of shared policies and strategies, a lack of leadership and conflicting historical professional roles and boundaries, amongst others.

With challenges from both the strategic and local level, it appears that the implementation of policies such as CPA will struggle from the outset unless the issues identified in this study are addressed.

## Implications for integrated care

Within the UK and across the world, governments have been driving forward changes to health and social care provision. Central to many of these policies has been the integration of services across all fields of practice. However, the process of providing integrated care through historically divided organisations has repeatedly been shown to be difficult with clients regularly falling between the two proverbial stools of health and social services.

The process of integration is extremely complex and requires organisations to consider not only its external but also its internal processes and relationships. For policies such as CPA where integrated working is central to its success, and at a time when such policies requiring integration are increasingly common there needs to be a team and an environment which is able to take into account, and address, the factors identified in this study. Without such forward thinking it is likely that such integrated policies will struggle at the implementation stage and in many cases may fail to achieve that which they set out to do.

The results presented in this review are based on the author's theses presented at the Royal College of Nursing Research Conference (Belfast, Northern Ireland) on 13 March 2013.

## Further articles by this author

Kelly M, Humphrey C. Implementation of the care programme approach across health and social services for dual diagnosis clients. Journal of Intellectual Disabilities 2013;17(4):314–28. Available from: http://jid.sagepub.com/content/early/2013/10/16/1744629513508383.full.pdf

## Further reading:

Goodwin N, Lawton-Smith S. Integrating care for people with mental illness: the Care Programme Approach in England and its implications for long-term conditions management. International Journal of Integrated Care 2010; 10(1). Available from: http://www.ijic.org/index.php/ijic/article/view/URN%3ANBN%3ANL%3AUI%3A10-1-100855/1033

